# Characterising risk of non-steroidal anti-inflammatory drug-related acute kidney injury: a retrospective cohort study

**DOI:** 10.3399/BJGPO.2021.0208

**Published:** 2022-01-12

**Authors:** Sharon X Lin, Thomas Phillips, David Culliford, Christopher Edwards, Christopher Holroyd, Kinda Ibrahim, Ravina Barrett, Clare Howard, Ruth Johnson, Jo Adams, Mathew Stammers, Adam Rischin, Paul Rutter, Nicola Barnes, Paul J Roderick, Simon DS Fraser

**Affiliations:** 1 National Institute for Health Research Wessex Applied Research Collaboration, University of Southampton, Southampton General Hospital, Southampton, UK; 2 University Hospital Southampton NHS Foundation Trust, Southampton General Hospital, Southampton, UK; 3 Clinical Research Network Wessex, Southampton, UK; 4 School of Pharmacy and Biomolecular Sciences, Cockcroft Building, University of Brighton, Brighton, UK; 5 Wessex Academic Health Science Network, Southampton Science Park, Southampton, UK; 6 Commissioning Support Unit, NHS South, Central and West, Hampshire, UK; 7 Health Sciences, University of Southampton, Southampton, UK; 8 Alfred Health, Melbourne, Australia; 9 School of Pharmacy and Biomedical Sciences, University of Portsmouth, Portsmouth, UK; 10 School of Primary Care, Population Sciences and Medical Education, Faculty of Medicine, University of Southampton, Southampton General Hospital, Southampton, UK

**Keywords:** primary health care, acute kidney injury, large database research, anti-inflammatory agents, non-steroidal

## Abstract

**Background:**

Non-steroidal anti-inflammatory drugs (NSAIDs) are commonly prescribed for pain and inflammation. NSAID complications include acute kidney injury (AKI), causing burden to patients and health services through increased morbidity, mortality, and hospital admissions.

**Aim:**

To measure the extent of NSAID prescribing in an adult population, the degree to which patients with potential higher risk of AKI were exposed to NSAIDs, and to quantify their risk of AKI.

**Design & setting:**

Retrospective 2-year closed-cohort study.

**Method:**

A retrospective cohort of adults was identified from a pseudonymised electronic primary care database in Hampshire, UK. The cohort had clinical information, prescribing data, and complete GP- and hospital-ordered biochemistry data. NSAID exposure (minimum one prescription in a 2-month period) was categorised as never, intermittent, and continuous, and first AKI using the national AKI e-alert algorithm. Descriptive statistics and logistic regression were used to explore NSAID prescribing patterns and AKI risk.

**Results:**

The baseline population was 702 265. NSAID prescription fell from 19 364 (2.8%) to 16 251 (2.4%) over 2 years. NSAID prescribing was positively associated with older age, female sex, greater socioeconomic deprivation, and certain comorbidities (diabetes, hypertension, osteoarthritis, and rheumatoid arthritis) and negatively with cardiovascular disease (CVD) and heart failure. Among those prescribed NSAIDs, AKI was associated with older age, greater deprivation, chronic kidney disease (CKD), CVD, heart failure, diabetes, and hypertension.

**Conclusion:**

Despite generally good prescribing practice, NSAID prescribing was identified in some people at higher risk of AKI (for example, patients with CKD and older) for whom medication review and NSAID deprescribing should be considered.

## How this fits in

NSAIDs are prescribed and taken ‘over the counter’ for pain and inflammation. Many NSAID risks, including gastrointestinal bleeding and AKI, are well documented and there are clear prescribing guidelines to reduce NSAID-related harm. However, there remains some NSAID prescribing among people at high AKI risk. Quantifying the AKI risk associated with NSAIDs in different groups and examining NSAID prescribing patterns would be useful to guide practice. This study used 2 years’ data from a large primary care database in Hampshire to explore NSAID prescribing practice and associated AKI risk. The study found that while NSAID prescribing generally declined over a 2-year period and NSAIDs were prescribed less frequently in at-risk groups, such as those with CVD and heart failure, some NSAID prescribing among people at risk remained, and this was associated with greater AKI risk. Older people, those living in more socioeconomically deprived areas, and those with comorbidities (including CKD, CVD, heart failure, diabetes, and hypertension) were at higher AKI risk.

## Introduction

NSAIDs are prescribed or bought ‘over the counter’ for a wide range of conditions and have analgesic and anti-inflammatory effects through inhibition of cyclooxygenase (COX)-1 and COX-2 enzymes, which are involved in prostaglandin synthesis.^1^ An estimated 6 million people aged >65 years in the UK suffer from a musculoskeletal problem such as osteoarthritis.^2,3^ Safe pharmacological pain relief options for these conditions are limited and NSAIDs are often prescribed.^4^ NSAID safety concerns, especially for older people, are known and their complications well described, the risk of which increase with age, dose, and duration of use.^5–8^ In a multimorbid, ageing population, complications, such as bleeding, AKI, worsening CKD, worsening heart failure, anaemia, myocardial infarction, and stroke, are key risks that lead to additional morbidity burden for patients, mortality, and high health service use.^1^ All NSAIDs increase risk of adverse events, although there is variation in side-effect risk profile. For example, COX-2 selective inhibitors and diclofenac are associated with higher risk of thrombotic events than naproxen, and piroxicam is associated with higher gastrointestinal risk than ibuprofen.^6,9^ A systematic review and meta-analysis of five observational studies exploring risk of individual NSAID-related AKI risk did not identify a significant risk difference between specific agents.^10^ AKI risk is common, particularly among older people and those with certain long-term conditions, such as CKD and heart failure, and is associated with several adverse outcomes, including worsening of CKD, higher healthcare costs, hospital admission, and mortality.^7^ More recent work reaffirms the association between NSAID prescribing and AKI risk in people aged ≥60 years in both hospital and primary care settings.^11^

The Medicines and Healthcare products Regulatory Agency (MHRA) and the National Institute for Health and Care Excellence (NICE) have published guidance on NSAID use, and NSAIDs are included in the American Geriatric Society Beers Criteria for potentially inappropriate medication for use in older adults.^8,9,12^ Despite awareness of risk among clinicians and presence of this guidance, NSAID use is still common, perhaps in part owing to the limited pain-relief options available.^5^ In the population-representative Health Survey for England 2016, 11% of the total population were taking some form of analgesia or NSAID.^13^ Self-medication with NSAIDs is also common; for example, in a Dutch study, about 30% of the general population sample and 13% of a ‘high-risk’ sample reported NSAID use within the preceding 4 weeks.^14^ Around 90 000 hospital admissions in England each year are related to adverse drug reactions and approximately 30% of those in older people are thought to be owing to NSAIDs, most commonly gastrointestinal bleeding, but renal impairment is also an important cause.^15,16^

There is some evidence of a changing pattern of NSAID prescribing over time, with reduction in certain key groups in which there are safety concerns, probably relating to MHRA and NICE guidance to GPs.^17,18^ The PINCER trial demonstrated that a pharmacist-led intervention was effective at reducing non-selective NSAID prescribing in people with a history of peptic ulcer without co-prescription of a proton pump inhibitor.^19^ National roll out of the PINCER intervention in the UK has led to demonstrable reductions in NSAID prescribing to those at risk of gastrointestinal bleeding.^20^ However, the extent of NSAID prescribing for groups at higher risk of non-gastrointestinal adverse outcomes, such as AKI, is not so well described. Identifying those at high risk would further inform NSAID medicines optimisation and deprescribing endeavours. The aim of this study was to establish the extent of NSAID prescribing in a large primary care population, to identify the characteristics of those at high risk of AKI, and to quantify their AKI risk in order to address the concern of ongoing risk of AKI among people still being prescribed NSAIDs.

## Method

### Population

The study population was drawn from the Care and Health Information Analytics (CHIA) database. This is a pseudonymised electronic database containing linked primary care data for approximately 1.4 million patients across Hampshire, UK, and clinical biochemistry results (only using creatinine and AKI e-alert data for the purpose of this study) from two large hospital laboratories (University Hospital Southampton NHS Foundation Trust and Portsmouth Hospitals University NHS Trust). A 2-year (1 October 2017–30 September 2019) retrospective cohort of individuals aged ≥18 years — for whom complete GP-ordered tests and all hospital-ordered tests were available for the duration of the study — was used to explore the relationship between NSAID exposure and AKI alerts (both defined below).^21^ The study population consisted of adults registered with GP practices in Hampshire that consistently sent all laboratory data to one of the two hospitals (Southampton or Portsmouth) for the duration of the study (in order to allow capture of all AKI alerts) and who were alive on 1 October 2017 and survived until 30 September 2019. It was therefore a ‘closed’ cohort. ‘Baseline’ was defined as the first 2-month period of the study for the purposes of describing NSAID exposure.

### NSAID prescribing

NSAID exposure was defined as having at least one primary care issued prescription of any oral NSAID (any drug within *British National Formulary* section 10.1.1: ‘Non-steroidal anti-inflammatory drugs’) recorded in a 2-month period.^22^ Two-month periods were chosen because repeat prescribing in the UK commonly adopts a 1- or 2-month prescription pattern. It was not possible to assess ‘over-the-counter’ NSAID consumption as pharmacy sales data were not available in CHIA. NSAID exposure was therefore characterised based on prescriptions issued as ‘never prescribed’ (no NSAID prescriptions in the entire 2-year study period), ‘single prescription only’ (any one NSAID prescription in any 2-month period of the 2-year study period), ‘multiple prescriptions in the prevalent NSAID group’ (>1 NSAID prescription in any 2 months of the 2-year study period in those who were prescribed NSAID at baseline), or ‘multiple prescription in the incident NSAID group’ (>1 NSAID prescription in any 2 months of the 2-year study period in those who were not prescribed NSAID at baseline). Age was defined in years, which was based on birth date before pseudonymisation (full date of birth was not available to the study team). Ethnic group, where available, was categorised into the following: British and Mixed British (Irish, Other, or White); Mixed (White and Asian, White and Black African, White and Black Caribbean, or Other Mixed); Indian, Bangladeshi, Pakistani, or Other Asian; African, Caribbean, or Other Black; Other; and Missing). Socioeconomic status was defined using the 2019 Index of Multiple Deprivation (IMD) quintiles.^23^ The IMD is a small-area measure of socioeconomic status, ranked nationally, and comprises the following seven domains: income; employment; education, skills, training; health and disability; crime; barriers to housing and services; and living environment. Baseline comorbidities were defined as ever having had a diagnosis of the condition coded in the primary care record using a set of standard Read codes (used to record primary care diagnoses in England), as described in a previous article using CHIA data.^21^ Particular groups potentially at increased risk from NSAID-related AKI were identified, including CKD (excluding kidney dialysis and transplant), CVD (including cerebrovascular disease, ischaemic heart disease, and peripheral vascular disease), heart failure (including cor-pulmonale), and older people (classified as aged ≥60 years). In addition, the number and proportion of each risk group prescribed NSAIDs in each 2-month period of the study period were identified and described.

### AKI risk

In the UK, the NHS implemented an AKI detection algorithm, based on the now widely used Kidney Disease: Improving Global Outcomes (KDIGO) creatinine change criteria, which generated electronic AKI alerts (e-alerts) for clinicians.^24^ This algorithm was used by the data provider before data extraction to identify AKI-alert occurrences in the cohort. Read codes for AKI were also identified and the main study outcome was AKI risk, defined as the rate of AKI occurrences in relevant sample populations. AKI in this study was defined as first occurrence of any AKI alert or Read code (used to record primary care diagnoses)^24^ during the study period, regardless of AKI stage.

### Statistical analyses

Descriptive statistics were used to identify the prevalence of NSAID exposure in the whole cohort and the defined subgroups at baseline and in each 2 months of the study period. Other medications were not considered (see ‘Strengths and limitations’ section). The numbers and proportions of people having at least one AKI alert during the 2-year period, as well as within 4 months from the start of a prescription, were described by NSAID exposure category (not prescribed, one prescription only, and multiple prescriptions with and without a baseline prescription). Univariate logistic regression was used to explore baseline associations between patient characteristics and NSAID prescription. Multivariable logistic regression models were then fitted to assess the associations between NSAID exposure in each 2-month prescribing period and first AKI alert occurring either in the same or subsequent 2-month period. This was in order to capture NSAID exposure and AKI risk outcome for all of the differing patterns of NSAID exposure.

Finally, absolute risk of first AKI was calculated for people aged ≥60 years, with and without exposure to NSAIDs (either single or multiple prescriptions across the 2 years) and other key risks (being older, lower socioeconomic status, and presence of comorbidities). For this calculation, first AKI occurring at any point across 2 years was used to calculate AKI risk in their respective sample population. This was to reflect the real-world possibility of people with any prescription of NSAIDs in a given period taking them sporadically rather than exactly as prescribed.

## Results

**Figure 1.**
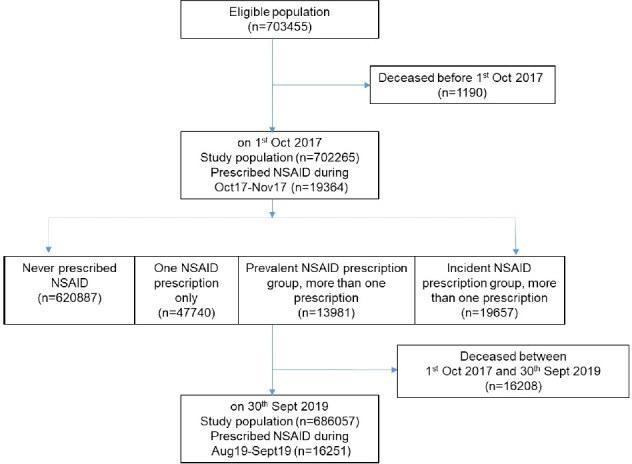
Flow diagram of study participants. NSAID = non-steroidal anti-inflammatory drug.

The closed study population comprised 702 265 people registered with 85 GP practices at study baseline (Figure 1). Mean age was 52 years and 53.2% were female, 14.6% lived in the most deprived quintile of IMD, and the majority were of British and Mixed British ethnic group, although 35.0% of individuals had no ethnic group data recorded (see Table 1). The most common long-term conditions were hypertension and osteoarthritis, and 5.1%, 9.5%, and 1.8% of the population had a history of CKD, CVD, and heart failure, respectively.

**Table 1.** Cohort characteristics at baseline

Characteristic		*n*	**%^a^**
Age, years	Median (IQR)	52	36–66
	Mean (SD)	52	19
Age group, years	18–39	210 047	29.9
	40–59	241 831	34.4
	60–79	194 168	27.6
	≥80	56 219	8.0
Sex	Male	328 712	46.8
	Female	373 553	53.2
Socioeconomic status (IMD quintile)	1 (most deprived)	102 796	14.6
	2	126 069	18.0
	3	132 262	18.8
	4	146 622	20.9
	5 (least deprived)	186 284	26.5
	Missing	8232	1.2
Ethnic group	British and Mixed British (Irish, Other, or White)	427 019	60.8
	Mixed (White and Asian, White and Black African, White and Black Caribbean, or Other Mixed)	3863	0.6
	Indian, Bangladeshi, Pakistani, or Other Asian	13 180	1.9
	African, Caribbean, or Other Black	5251	0.7
	Other	7236	1.0
	Missing	245 716	35.0
Long-term condition diagnosed at the point of baseline	Chronic kidney disease	36 059	5.1
	CVD (ischaemic heart disease, cerebrovascular disease, and peripheral cardiovascular disease)	52 985	7.5
	Heart failure	12 799	1.8
	Diabetes	62 767	8.9
	Hypertension	150 458	21.4
	Osteoarthritis	95 294	13.6
	Rheumatoid arthritis	6723	1.0
Prescribed at baseline	NSAID	19 364	2.8

^a^Unless otherwise stated. CVD = cardiovascular disease. CVD = cardiovascular disease. IQR = interquartile range. IMD = Index of Multiple Deprivation. NSAID = non-steroidal anti-inflammatory drug. SD = standard deviation.

### NSAID prescription

**Figure 2.**
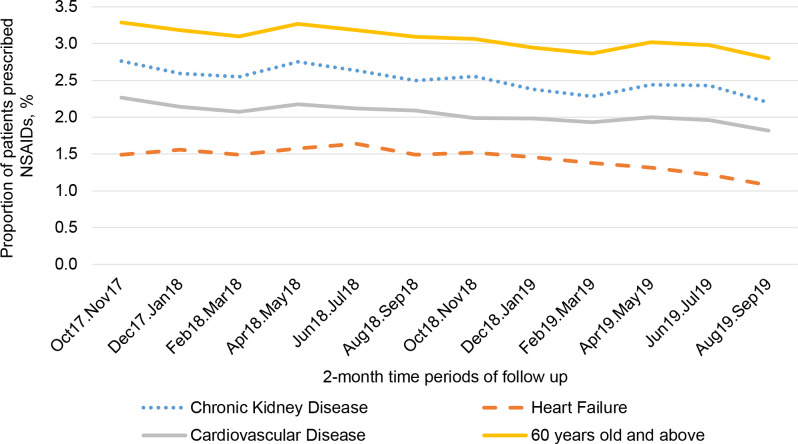
Proportion of patients in each of the risk groups prescribed NSAIDs in each 2-month time period of follow up

A total of 19 364 (2.8%, *n* = 702 265) people were prescribed oral NSAIDs at baseline (Table 1). This had fallen to 16 251 (2.4%, *n* = 686 057) excluding the deceased during the 2-year study perod (Figure 1). During the 2-year follow-up period, 620 887 (88.4%) were never prescribed NSAIDs at any point (Figure 1, Supplementary Table S1), 47 740 (6.8%) were prescribed NSAIDs once (single NSAID group), 13 981 (2.0%) were prescribed NSAIDs multiple times, including a baseline prescription (prevalent NSAID group), and 19 657 (2.8%) were also prescribed NSAIDs multiple times, but not at baseline (incident NSAID group). In the groups at potentially higher risk of AKI (older age [aged ≥60 years], CKD, CVD, and heart failure), NSAID prescribing fell over the study period (Figure 2); for example, among people with CKD, baseline prescription rate of 2.8% fell to 2.2% at the end of the study period. The total number of patients with heart failure, CVD, or CKD prescribed >1 NSAID remained 4686 (see Supplementary Table S1). NSAID prescription at baseline was associated with older age, female sex, greater socioeconomic deprivation, history of diabetes, hypertension, osteoarthritis, and rheumatoid arthritis (Table 2). Those with CVD (odds ratio [OR] 0.80, 95% confidence interval [CI] = 0.76 to 0.85, *P*<0.001) or heart failure (OR 0.51, 95% CI = 0.44 to 0.59, *P*<0.001) were less likely to be prescribed NSAIDs, and there was no association (either positive or negative) with having CKD (OR 1.00, 95% CI = 0.94 to 1.06, *P* = 0.96).

**Table 2.** Univariate associations with NSAID prescription at baseline

Characteristic		Odds ratio^a^	95% CI		*P*-value
Age group, years (versus 18–40)	40–59	2.20	2.11	2.29	0.02
	60–79	2.59	2.48	2.71	<0.001
	≥80	1.29	1.20	1.39	<0.001
Sex (versus male)	Female	1.22	1.19	1.26	<0.001
Socioeconomic status, IMD quintile (versus 5, least deprived)	4	1.06	1.01	1.10	<0.001
	3	1.15	1.10	1.20	<0.001
	2	1.29	1.23	1.35	<0.001
	1 (most deprived)	1.54	1.47	1.61	<0.001
CKD (versus no CKD)		1.00	0.94	1.06	0.96
CVD (versus no CVD)		0.80	0.76	0.85	<0.001
Heart failure (versus no heart failure)		0.51	0.44	0.59	<0.001
Diabetes (type 1 or 2 versus no diabetes)		1.31	1.26	1.37	<0.001
Hypertension (versus no hypertension)		1.44	1.39	1.49	<0.001
Osteoarthritis (versus no osteoarthritis)		2.82	2.73	2.91	<0.001
Rheumatoid arthritis (versus no rheumatoid arthritis)		5.08	4.71	5.47	<0.001

^a^For those with multiple categories odd ratios were compared with base groups. CKD = chronic kidney disease. CVD = cardiovascular disease. IMD = Index of Multiple Deprivation. NSAID = non-steroidal anti-inflammatory drug.

### AKI risk

**Figure 3.**
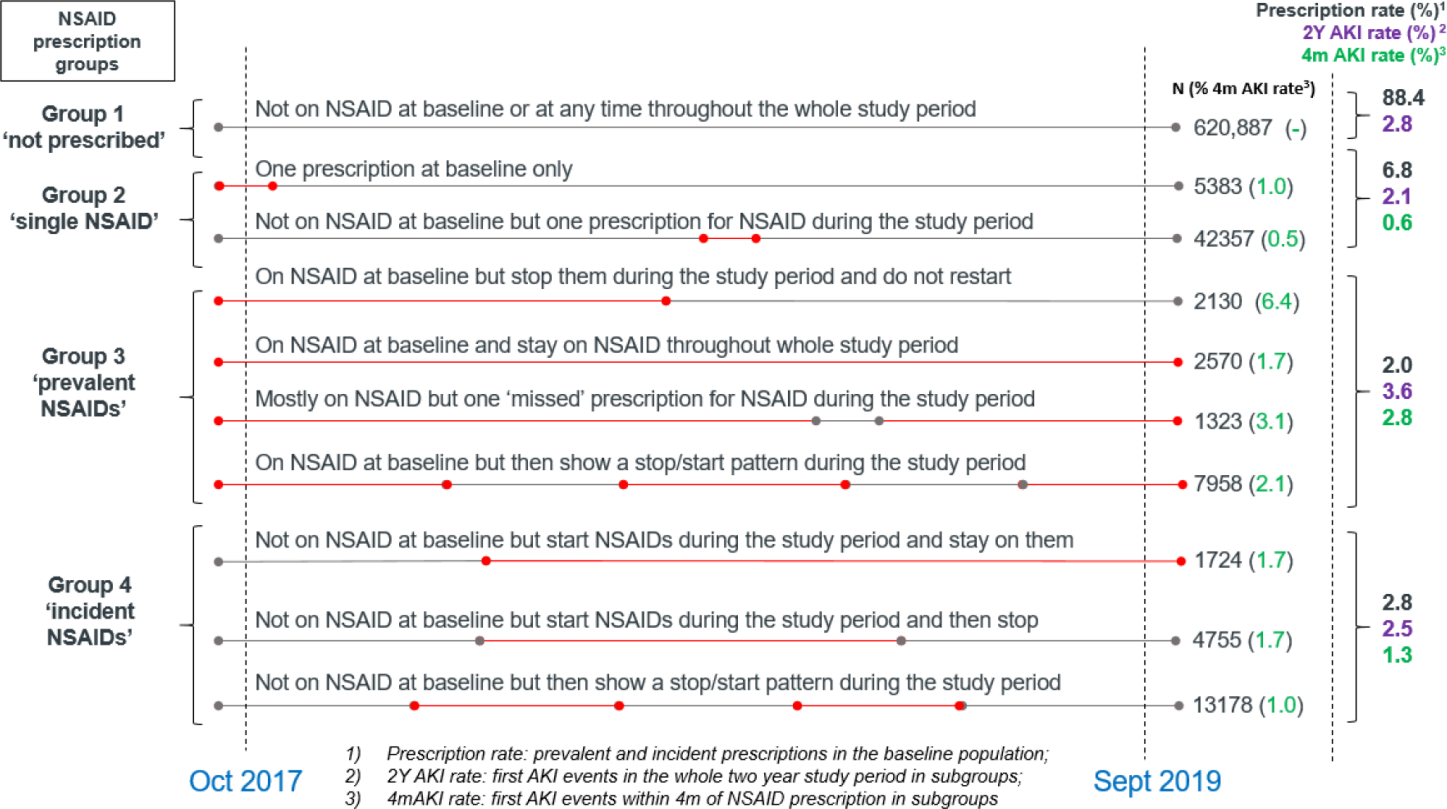
Non-steroidal anti-inflammatory drug (NSAID) prescription patterns throughout the study period. AKI = acute kidney injury. M = month. Y = year.

The highest proportion of first AKI alert, hence AKI risk in NSAID prescription groups, was 3.6% in the ‘prevalent NSAID group’ (Figure 3), followed by 2.8% in the ‘no prescription group’, 2.5% in the ‘incident NSAID group’ and 2.1% in the ‘single prescription group’. The same order of AKI risk was also found in the subgroups within 4 months of prescription: 2.8%, 1.3%, and 0.6% (by definition the group not prescribed NSAID did not have a value). The proportion of people with multiple AKI alerts during the 2-year follow up was very similar in each NSAID exposure category (see Supplementary Figure S1).

### Associations with AKI risk

Among those prescribed NSAIDs at any point (single, prevalent, and incident NSAID groups) through the study the following was found: people of older age groups experienced between 65% (OR 1.65, 95% CI = 1.27 to 2.18, *P*<0.001) and 657% (OR 7.57, 95% CI = 5.53 to 10.42, *P*<0.001) more risk of AKI alerts within 4 months of prescription than the youngest group; those with greater social deprivation were more likely to have AKI alerts (19%–69%); and the disease group with history of CKD, CVD, heart failure, diabetes, and hypertension were 41%-78% more likely to have AKI alerts (Table 3). No significant differences were present between male and female groups. Absolute risk of first AKI was highest among those aged ≥80 years with greater combinations of hypertension, diabetes, CKD, CVD, and heart failure, and those from lower socioeconomic groups (see Supplementary Table S2 and Supplementary Figure S2).

**Table 3.** Multivariate logistic model on associations between NSAID prescription and AKI within 4 months from the start of prescription period

Characteristic		Odds ratio	95% CI		*P*-value
Age group, years (versus 18–40)	40–59	1.65	1.27	2.18	<0.001
	60–79	3.98	3.07	5.22	<0.001
	≥80	7.57	5.53	10.42	<0.001
Sex (versus male)	Female	1.11	0.97	1.27	0.13
Socioeconomic status, IMD quintile (versus 5, least deprived)	4	1.19	0.96	1.46	0.11
	3	1.29	1.04	1.59	0.02
	2	1.64	1.34	2.01	<0.001
	1 (most deprived)	1.69	1.36	2.09	<0.001
CKD (versus no CKD)		1.61	1.30	1.97	<0.001
CVD (versus no CVD)		1.78	1.47	2.14	<0.001
Heart failure (versus no heart failure)		1.78	1.23	2.51	0.001
Diabetes (type 1 or 2 versus no diabetes)		1.50	1.25	1.78	<0.001
Hypertension (versus no hypertension)		1.41	1.21	1.64	<0.001

Cohort: *N* = 77 422; AKI: *n* = 915. CKD = chronic kidney disease. CVD = cardiovascular disease. IMD = Index of Multiple Deprivation.

## Discussion

### Summary

This retrospective cohort study identified the extent and distribution of NSAID prescribing and estimated the risk of AKI in a large primary care population. There were indicators of good practice in concordance with new guidance on NSAID prescribing. Overall, fewer patients in high-risk groups received these medications and the incidence of prescribing fell during the study period (Figure 2), despite natural ageing of the population in this closed cohort. Patients with known risk factors of AKI, particularly in the context of NSAID prescribing, such as CVD and heart failure, were all prescribed NSAIDs less frequently than those without those risk factors. However, there was still an important minority of those with known risk factors, including heart failure, CVD, or CKD who were prescribed NSAIDs. The lack of association between NSAID prescribing and having CKD may be a concern. The association was not negative as it was for CVD and heart failure, which may suggest that patients having CKD is not as strong a deterrent for NSAID prescribing by GPs as CVD or heart failure.

Established risk factors for AKI were also reflected in this dataset; for example, older age, lower socioeconomic status, CKD, CVD, heart failure, diabetes, and hypertension all showed increased AKI rates within 4 months of NSAID prescription. The cohort identified NSAID prescription at baseline positively associated with older age, female sex, greater socioeconomic deprivation, history of diabetes, hypertension, osteoarthritis, and rheumatoid arthritis potentially reflecting greater need for pain relief in these groups. The authors suspect (although did not demonstrate) that people with diabetes and hypertension were also more likely to be co-prescribed renin-angiotensin system inhibitors or diuretics, which may further increase the risk of AKI.^25,26^

The development of an NSAID-related AKI risk tool was explored, based on absolute risk of first AKI among those of older age, lower socioeconomic status, and combinations of comorbidities (hypertension, diabetes, CKD, CVD, and heart failure). This pointed to the potential value of identifying those with higher risk combinations of these exposures in informing deprescribing endeavours, but further work is needed to validate this risk tool (see Supplementary Figure S2). For example, the calculation of absolute risk was limited by the lack of knowledge about when exactly people took NSAIDs.

### Strengths and limitations

The strengths of this study include a large primary care cohort with detailed NSAID prescribing data and its linked hospital and GP biochemical tests including creatinine, which enabled the characterisation of NSAID exposure and AKI risk.

Limitations of this data were an inability to identify and track over-the-counter NSAID sales and use. Data were also not able to be captured on patients who may vary the use of NSAIDs or stop and start them according to symptoms, or continue at a lower dose. Some people would have moved out of the area during the study period, which means their data would no longer be captured and may therefore potentially decrease the prescription rate and death rate in this closed cohort. There was potential for further confounding arising from exposures that were unable to be considered, including other comorbidities and medications, such as renin-angiotensin system inhibitors and diuretics. Individual NSAIDs were not specified, although a recent review shows no appreciable AKI risk difference between agents.^10^ Other medications were not considered in this study, which might introduce additional risk. There was also a high proportion of missing ethnic group data, which hampers broader application of this data in different subpopulations and conclusions as to ethnic group as a non-modifiable risk factor. The baseline comorbidities were recorded to include all historic, therefore some may no longer be currently ‘active’. Severe AKI was not explored, which is relatively common in people with a history of CKD. In the study, 6400 had more severe AKI (see Supplementary Table S3) and it would be useful to support future work between NSAID and severity of AKI. Data were limited in geography to the south of England. In other settings, such as areas of greater deprivation or those with higher rates of CVD and/or CKD, there may be larger numbers of patients at risk.

### Comparison with existing literature

Similarly to the present study's findings, a Scandinavian study investigating NSAIDs in patients with CVD showed that prescribing was still as high (around 10%), but has been decreasing with time. Diabetes and hypertension also predispose to higher NSAID prescription rates in this study.^27^ Over-the-counter sales of available NSAIDs in the region have also been noted to have increased.^28^ Another European study identified a fall in diclofenac prescribing after regulatory advice was issued, but the gradient of this reduction was slowing in more recent years, particularly in those with diabetes and hypertension.^29^

### Implications for research and practice

The practical implications of these findings are that it is still important to review NSAID prescribing, particularly for those in high-risk groups for AKI. It may be advisable to flag patients in these groups in primary care to avoid or reduce the number of prescriptions. Further exploration would be valuable to the process of initiation and review of NSAID prescriptions during clinical consultations and medication reviews. It would also be valuable to investigate the extent to which an AKI alert leads practitioners to cease NSAID prescription. There is clearly a burden of musculoskeletal disease for which many still rely on NSAIDs, and alternative, effective medicines may be lacking. Non-pharmacological pain management strategies that address the whole person involving behaviour research could be explored. Topical NSAIDs have been shown to be associated with a lower risk of AKI than oral NSAIDs, even when the oral NSAIDs were short course only (analogous to the intermittent prescribing group).^11^ Changing to topical NSAIDs may represent one strategy for deprescribing or not initiating oral NSAID prescription among those at highest risk. Deprescribing NSAIDs has been shown to be pragmatic, cost-effective, and potentially beneficial to patients in other settings, as well as in the UK.^30,31^ Other pharmaceutical agents for pain relief or other alternative pain relief approaches need further exploration. Non-pharmacological pain management strategies that address whole-person needs require further exploration for the groups of patients who have been identified but are beyond the remit of this article.

In conclusion, this large retrospective cohort study, using routine primary care and laboratory data, identified that while NSAID usage was falling overall, some people in the population with high risk of developing AKI were still being prescribed NSAIDs and they were shown to be at greater risk of AKI. These findings support targeted NSAID deprescribing efforts in high-risk groups and highlight which patients are most at risk.
